# Review of menopausal palpitations measures

**DOI:** 10.1186/s40695-021-00063-6

**Published:** 2021-05-31

**Authors:** Ying Sheng, Janet S. Carpenter, Charles D. Elomba, Jennifer S. Alwine, Min Yue, Caitlin A. Pike, Chen X. Chen, James E. Tisdale

**Affiliations:** 1 School of Nursing, Indiana University, Indianapolis, IN 46202 USA; 2 College of Pharmacy, Purdue University, West Lafayette, IN 47907 USA; 3 University Library, Indiana University, Indianapolis, IN 46202 USA; 4 School of Medicine, Indiana University, Indianapolis, IN 46202 USA

**Keywords:** Menopause, Palpitations, Symptom assessment, Outcome assessment, Patient-reported outcome measures

## Abstract

Palpitations are reported commonly by women around the time of menopause as skipped, missed, irregular, and/or exaggerated heartbeats or heart pounding. However, much less is known about palpitations than other menopausal symptoms such as vasomotor symptoms. The objective of this review was to integrate evidence on menopausal palpitations measures. Keyword searching was done in PubMed, CINAHL, and PsycINFO for English-language, descriptive articles containing data on menopause and palpitations and meeting other pre-specified inclusion criteria. Of 670 articles, 110 met inclusion criteria and were included in the review. Results showed that 11 different measures were used across articles, with variability within and between measures. Inconsistencies in the wording of measurement items, recall periods, and response options were observed even when standardized measures were used. Most measures were limited to assessing symptom presence and severity. Findings suggest that efforts should be undertaken to (1) standardize conceptual and operational definitions of menopausal palpitations and (2) develop a patient-friendly, conceptually clear, psychometrically sound measure of menopausal palpitations.

## Introduction

Palpitations are more common in women than in men [1]. Women may experience palpitations during peri- and post-menopause, along with vasomotor and other menopausal symptoms [1–3]. Although much has been written about menopausal vasomotor symptoms [4, 5], less has been reported about menopausal palpitations. Menopausal women report feeling skipped, missed, irregular, and/or exaggerated heartbeats or heart pounding [6, 7]. A recently completed review found that palpitations prevalence was 4 to 40% in premenopausal women, 20 to 40% in perimenopausal women, and 16 to 54% in postmenopausal women [8]. The review also showed that the prevalence was significantly higher among perimenopausal and surgically postmenopausal women in comparison to premenopausal or postmenopausal women [8]. Although the number of articles reviewed was small (*n* = 5), differences in palpitations assessment tools limited the ability to make comparisons across articles.

Palpitations are associated with some negative health outcomes in the general population and in menopausal women. In the general population, palpitations (1) account for 16% of general physicians’ visits [9], (2) are the second leading reason for cardiologist visits [9], and (3) are related to increased risk for cardiac arrythmias when they affect sleep and work [10]. In peri- and postmenopausal women, menopausal palpitations distress is associated with worse sleep disturbance [11], depressive symptoms [3, 11, 12], stress [11], and menopausal quality of life [11]. The directions of these associations are unclear as these were cross-sectional analyses. Comprehensive assessment methods are critical to understanding palpitations and improving menopausal quality of life.

There do not appear to be any existing published reviews of menopausal palpitations measures. It is important to understand how palpitations have been measured. First, palpitations are likely experienced by women globally during their transition through menopause [8]. Using the same measure of palpitations can facilitate comparisons across studies to help understand inter-individual differences in symptoms by race, ethnicity, geographic region, and other cultural variables [13]. Second, examining current measures of palpitations can help to explicate what symptom dimensions have and have not been explored. The Theory of Unpleasant Symptoms posits that symptoms can be measured on multiple dimensions [14]. Symptom dimensions that can yield important information for clinical practice and research include presence, frequency, severity or intensity, bother or distress, interference or impact, temporal pattern, duration, quality, degree of unpredictability, perceived control over, and symptom representations. More frequent palpitations may be associated with serious arrhythmias in the general population [15], which can indicate serious cardiovascular disease. In the absence of cohesive information regarding palpitations measures, it is unclear whether and to what degree various symptom dimensions are being assessed and reported. The purpose of this review was to integrate evidence on menopausal palpitations measures.

## Methods

### Eligibility criteria

The review was conducted with pre-specified inclusion criteria. Articles had to be 1) full-length, 2) peer-reviewed, 3) descriptive research, 4) English-language, 5) conducted in menopausal women (samples described as midlife, menopausal, peri- and/or post-menopausal consistent with Stages of Reproductive Aging Workshop (STRAW) or STRAW+ 10 definitions) [16–18], and 6) inclusive of data on menopausal palpitations or similar symptoms such as racing or pounding heart. We selected descriptive research to be consistent with current recommendations for excluding clinical trials in measurement-focused reviews [19]. Articles that included premenopausal women as a comparison group were included but articles that focused exclusively on premenopausal women were excluded. Other exclusion criteria were articles that 1) included transgender or gender transitioning populations, men, or animals, or 2) defined study populations as “menopausal women” or “symptomatic women” without additional information.

### Literature search strategy

A librarian (CAP) completed the search on May 19, 2020 with no restriction on publication date to reflect all available published articles up to that date. PubMed, Cumulated Index to Nursing and Allied Health Literature (CINAHL), and PsycINFO search engines were selected because of their comprehensiveness and usefulness for health-related literature. Search strategy included the MeSH terms and key words: (“Menopause” OR menopaus*) AND (palpitation* OR heart racing OR heart pounding OR irregular heart). The search term “palpitations” was too specific to locate pertinent articles. Additional searches were done by searching for articles that employed menopausal-symptom assessment tools from the three search engines. Tools searched included the Menopause Rating Scale (Heinemann) [20], Greene Climacteric Symptom Rating Scale [21], Midlife Women’s Symptom Index [22], Holte/Mikkelsen Menopause Checklist [23], Hunter’s Women’s Health Questionnaire [24], Neugarten and Kraines’ Symptom Checklist [25], Study of Women’s Health Across the Nation (SWAN) menopausal symptom checklist, Menopause Symptoms List [26], the Blatt Kupperman Index [27–29], Menopause Symptoms Checklist [26], and Menopausal-Specific Quality of Life (MENQOL) [30].

We organized the review using Covidence.org, a structured program available through a university subscription. We used Covidence features to remove duplicates from searches, track the progress of multiple raters during screening and full-text review, and calculate inter-rater reliabilities. A separate literature search or review protocol was not published. The review did not meet the definition of human subject research and did not require institutional review board approval.

### Screening process

After completing the searches, one author deduplicated the list of potential articles. Next, article titles and abstracts (*n* = 670) were independently and sequentially screened by two of three authors. To be overly inclusive, screeners included articles if titles and abstracts mentioned (1) menopausal or climacteric symptoms/syndrome or (2) one of the aforementioned menopausal symptom assessment tools as a study measure. Where disagreements occurred (8.8%), the three reviewers discussed each article and achieved consensus.

Following an initial screening, there were 608 articles remaining, and full texts were retrieved by the team for review. Two authors independently and sequentially read each article and voted on their inclusion or exclusion. The data presentation for symptom surveys or the previously mentioned symptom assessment tools were carefully reviewed. Articles were excluded if there were no specific data on palpitations reported. Disagreements (5.0%) were again resolved through discussion.

### Data extraction process

Using data extraction forms that we created, one author extracted the data and two additional authors verified accuracy. Disagreements were resolved through discussion. Extracted data included article metadata (title, author, year, country, design) and palpitations measurement details (name, item, recall period, response options, symptom dimensions assessed). We did not assess quality and bias because the review was focused only on the measurement tool that was used and not the study sample, analysis or results.

In summarizing the data from the extraction process, we focused on number of articles and not the number of studies. Some author groups produced multiple articles from the same study but there were sometimes slight differences in sample sizes, characteristics, or data reported. For example, Blümel et al. (2011, 2012, 2016) [31–33] and Monterrosa, Blümet et al. (2007, 2009, 2013) [34–36] were from the multicentric Collaborative Group for Research of the Climacteric in Latin America (REDLINC) III, or IV, or V study. However, because our focus was on articles, we did not report these as one study or three waves of the same study because they had different samples, purposes, and/or data. Other authors produced multiple articles, but these were not always from the same study. For example, four articles by Chedrai [37–40] included Ecuadorian women but each article reflected a different sample. Thus, we use the term “articles” throughout this review rather than “studies”.

## Results

**Fig. 1:**
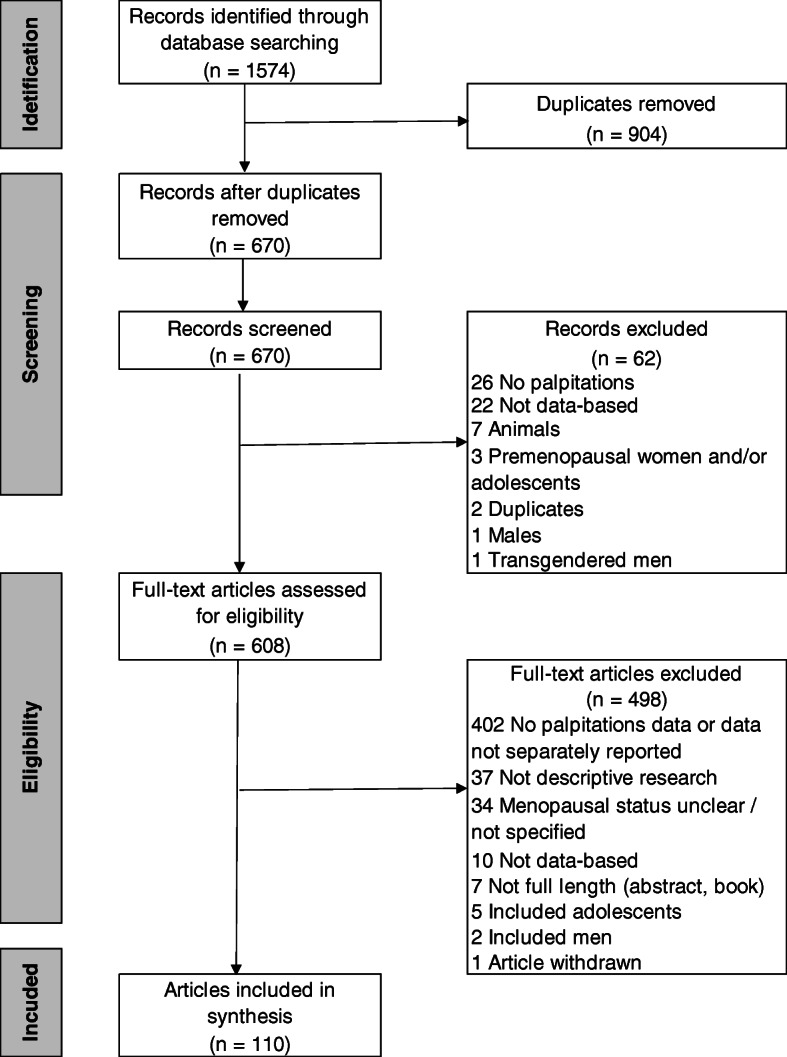
Flow diagram depicting disposition of the articles

The Preferred Reporting Items for Systematic Reviews and Meta-Analyses (PRISMA) flow diagram [41] is shown in Fig. 1. After searching the databases, 1574 articles were retrieved, leaving 670 articles after duplicates were removed. After screening titles and abstracts for the inclusion and exclusion criteria, 62 articles were excluded. Six hundred and eight full-text articles were screened, from which 498 were excluded because they did not report palpitations data; they were not descriptive research, data-based articles, or full-length articles; they included wrong sample; or the articles were withdrawn. This left 110 articles for the review.

### Measurement tools

Table 1 provides information regarding the different measurement tools used in the reviewed articles. Most articles used standardized instruments (76%) and the remaining articles used other measurement methods like unspecified self-administered questionaries or interviews (24%). The most common assessment method was the Menopause Rating Scale (*n* = 69) [31–40, 42–100]. The next three most common assessment methods were unspecified self-administered questionaries (*n* = 14) [115–128], interviews (*n* = 10) [7, 129–137], and the Blatt Kupperman Menopause Index (*n* = 8) [12, 44, 101–106]. With the exception of interviews, all measures used a single item to assess palpitations. In all articles, palpitations were measured along with other menopausal symptoms.

**Table 1:** Variability within and across measurement tools

Measure	Article n (citations)	Item	Recall period	Response options	
				Likert^a^	Yes/no^b^
**Standardized Questionnaires (*n*** **= 85)**^**c**^					
Menopause Rating Scale	45^c,d^ [31–40, 42–76]	Heart discomfort		5	
	5 [77–81]	Heart discomfort	1 week to 1 month	5	
	1 [82]	Heart discomfort			√
	1 [83]	Heart discomfort	1 month		√
	5 [84–88]	Heart discomfort/palpitation		5	
	1 [89]	Heart discomfort/palpitation	1 month		√
	7 [90–96]	Heart discomfort (unusual awareness of heartbeat, heart skipping, heart racing, tightness)		5	
	2 [97, 98]	Heart discomfort/cardiac symptoms		5	
	1 [99]	Cardiac symptoms (palpitations, racing, heartbeat, irregular beats, tightness in chest)		5	
	1 [100]	Cardiac		5	
Blatt-Kupperman Menopausal Index	6^c^ [12, 44, 101–104]	Palpitations		4	
	1 [105]	Palpitations			
	1 [106]	Rapid heartbeat		3	
Kaczmarek menopause-specific questionnaire	2 [107, 108]	Palpitations and butterflies			√
Simplified Menopause Index	1 [109]	Palpitation			
	1 [110]	Breathlessness and palpitations (I become short of breath and experience palpitations)			
Cardiovascular Symptom Index for Midlife Women	1 [111]	Palpitation		6	√
Menopausal-specific Quality of Life	1 [112]	Heart beating quickly and heart discomfort			√
Survey adapted from menopause symptom list	1 [113]	Palpitations (Heart beating quickly or strongly)		5	
Women’s Health Questionnaire	1 [114]	Tachycardia		4	√
**Other Assessment Tools (*n*** **= 26)**					
Unspecified self-administered questionnaire	4 [115–118]	Palpitation/heart palpitations			
	3 [119–121]	Palpitation			√
	1 [122]	Palpitation	1 year		√
	2 [123, 124]	Palpitation		4	
	1 [125]	Palpitations	1 year	4	
	1 [126]	Palpitation		4	
	1 [127]	Irregular heart beats			√
	1 [128]	Tachycardia			√
Interview	2 [129, 130]	Palpitations			
	1 [131]	Palpitations	1 month		√
	1 [7]^d^	Palpitations	1 month		
	1 [132]	Palpitations			√
	1 [133]	Palpitations	12 months	4 & 7^e^	√
	1 [134]	Palpitations	2 weeks		
	1 [135]	Heart pounding (or racing)	2 weeks		√
	1 [136]	Palpitation of heart, excitable/ anxiety			
	1 [137]	Rapid heartbeat			√^f^
Unspecified	2 [138, 139]	Palpitations		4	

Blank boxes = information was not reported in articles

^a^ Likert scale column denotes number of points on scale

• 7-point scale: how frequently experienced, details not specified

• 6-point scale: *0 no symptom to 5 extremely severe*

• 5-point scale variations were *0 not present/no symptoms/absent/no complaints, 1 mild, 2 moderate, 3 severe, 4 very severe/very severe complaints*; or *0.0 no symptom, 0.1–0.3 mild, 0.4–0.5 moderate, 0.6–0.7 severe, 0.8–1.0 very severe; or intensity of difficulties*; • *0 none, 1 minor, 2 medium, 3 major, 4 unbearable*

• 4-point scale variations were: *0 to 3 severity; Absent, low, moderate, strong; or 0 no, 1 slight, 2 moderate, 3 severe; or 0 no symptom to 3 severe; mild (once in a while) to very serious (requires treatment);* or *0 not at all, 1 sometimes, 2 often, 3 always; or 0 symptom did not occur, 1 mild symptom that did not interfere with usual life, 2 moderate symptom that interfered somewhat with usual activities,* • *3 severe symptom that prevented routine daily activities;* or *Grade 1 no symptoms, 2 slight symptoms, 3 moderate symptoms, 4 severe symptoms*

• 3-point scale: *1 slight symptoms, limited and not bothersome; 2 moderate symptoms, distressing, required medication but did not disrupt work, could be tolerated; 3 severe symptoms, distressing, required medication, and time off work*

^b^ Yes/no, presence/absence

^c^ One article used both Menopause Rating Scale and Blatt-Kupperman Menopause Index

^d^ Open-ended questions about severity

^e^ 4-point scale for distress and 7-point scale for frequency

^f^ Yes, no, and I don’t know

Table 1 also shows the wide variation in items, recall periods, and response options within and between the measures. The Menopause Rating Scale provides one example of measurement item variation. Rather than using the standardized item, articles reported the item as heart discomfort (with and without further specification), palpitations, or cardiac symptoms. Recall periods were mostly missing, and only 14 articles (12.7%) included recall periods. These reported recall periods ranged from 1 week to 1 year [7, 77–81, 83, 89, 122, 125, 131, 133–135]. Response options varied from yes/no to Likert scales. Most of the Likert scales measured symptom presence and severity (e.g., 0 no symptom, 1 mild, 2 moderate, 3 severe, 4 very severe) and only a few measured symptom frequency (e.g., 0 not at all, 1 sometimes, 2 often, 3 always) [12, 125].

### Concepts assessed by measurement tools

Table 2 maps the measurement items in Table 1 to various concepts being assessed including palpitations (not otherwise specified), sensations related to heart rate (e.g., heart racing), sensations related to heartbeat (e.g., heart pounding), and discomfort. None of the items had the same meaning or assessed the same concepts. What was assessed depended on the details of the items being used. For example, the underlying symptom concept assessed with heart discomfort (e.g., discomfort) was different from that assessed with an item of rapid heartbeat (e.g., sensations related to heartbeat).

**Table 2:** Concepts Assessed by Measurement Tools

Measurement Tool Item	Palpitations not specified	Heart rate sensations	Heartbeat sensations	Discomfort
Heart discomfort				X
Heart discomfort/palpitation	X			X
Heart discomfort (unusual awareness of heartbeat, heart skipping, heart racing, tightness)		X	X	X
Heart discomfort/cardiac symptoms				X
Cardiac symptoms (palpitations, racing heartbeat, irregular beats, tightness in chest)	X	X	X	
Palpitations/heart palpitations	X			
Palpitation of heart, excitable/anxiety	X			
Palpitations and butterflies	X		X	
Breathlessness and palpitations	X			
Palpitations (heart beating quickly or strongly)	X	X	X	
Irregular heart beats			X	
Tachycardia		X		
Rapid heartbeat		X		
Heart pounding (or racing)		X	X	
Heart beating quickly and heart discomfort		X		X

### Symptom dimensions assessed by measurement tools

Table 3 maps the measurement items in Table 1 to symptom dimensions of presence, severity (or intensity), distress (or bother), frequency, and interference (or impact). Most articles in this review used uni- or bi-dimensional measures (presence and/or severity). Two articles reported modifying bi-dimensional measures (presence, severity) based on participants’ feedback during pretesting [83, 89]. Likert response options were simplified to yes/no, making them uni-dimensional measures (presence) [83, 89]. Only seven (6.4%) articles measured dimensions such as distress/bother, frequency, or interference/impact of the palpitations [12, 104, 106, 113, 125, 126, 133]. Very few measures were multi-dimensional, and these assessed three or four symptom dimensions. No articles measured the following dimensions: temporal pattern, duration, quality, degree of unpredictability, perceived control over, or symptom representations.

**Table 3:** Symptom Dimensions Assessed by Measurement Tool^a^

Measurement Tool	Article n (citation)	Presence	Severity	Distress or Bother	Frequency	Interference or Impact
Menopause Rating Scale	66 [31–40, 42–81, 84–88, 90–100]	X	X			
	3 [82, 83, 89]	X				
Blatt-Kupperman Menopause Index	4 [44, 101–103]	X	X			
	1 [12]	X			X	
	1 [104]		X	X	X	X
	1 [106]		X	X		X
	1 [105]	–	–	–	–	–
Kaczmarek menopause-specific questionnaire	2 [107, 108]	X				
Simplified Menopause Index	2 [109, 110]	–	–	–	–	–
Cardiovascular Symptom Index for Midlife Women	1 [111]	X	X			
Menopausal-Specific Quality of Life	1 [112]	X				
Survey adapted from the menopause symptom list	1 [113]		X		X	
Women’s Health Questionnaires	1 [114]	X				
Unspecified self-administered questionnaire	2 [123, 124]	X	X			
	6 [119–122, 127, 128]	X				
	1 [125]	X			X	
	1 [126]	X	X			X
	4 [115–118]	–	–	–	–	–
Interview	1 [7]	X	X			
	4 [131, 132, 135, 137]	X				
	1 [133]	X		X	X	
	4 [129, 130, 134, 136]	–	–	–	–	–
Unspecified	2 [138, 139]	X	X			

Blank boxes = concept not assessed

-- Measurement details were not specified in article, concepts assessed are unclear

^a^ No articles addressed symptoms dimensions of duration / temporal pattern, degree of unpredictability, perceived control over, and symptom representations

## Discussion

Menopausal palpitations have been assessed using a variety of items on measures that were developed to assess multiple menopausal symptoms. This review documents the amount of variation in these measures’ items, recall periods, and response options both as they occur within a standardized measure and across standardized and unstandardized measures. In addition, this review documents the relatively limited way in which menopausal palpitations have been assessed to date using mostly single items with varying differentiation of concepts and a limited number of symptom dimensions.

There was a lack of conceptual and operational consistency identified in this review, making it impossible to provide a recommendation for which measure may be superior to others. The inconsistencies in items, recall periods, and response options indicated different concepts were assessed, which prohibits integrating research findings across articles. Variations were evident in whether palpitations were assessed as sensations related to heart beats, heart rate, discomfort, tightness, anxiety, and/or breathlessness. The words used to assess menopausal palpitations were numerous and included some that participants with low health literacy may not understand (i.e., palpitations, tachycardia). In addition, the few articles that provided a recall period indicated the symptom was assessed using different recall time frames. Assessing symptoms in the past week could lead to more accurate recall but would omit data about prior history of palpitations, whereas assessing over the past year could lead to imprecise estimations or memory recall bias but more historical information [140]. Measuring palpitations over a variety of time periods may provide the most comprehensive information. Furthermore, although most response options were presence and/or severity, the descriptions varied. For example, in 5-point response options, the meaning of 0 varied from no symptom to no discomfort and the meaning of upper scores varied from very severe symptom to unbearable or severe discomfort. The response options determine the way participants interpret and respond to the question [140]. It is possible that some participants may have had palpitations but did not experience discomfort from them, and thus, might have rated *0 no discomfort* or *1 symptom present* depending on how the item was worded. The variation in how palpitations are assessed limits the consistency and comparability of findings across articles.

It is impossible to differentiate how much of the operational and conceptual inconsistency was due to how the item was presented to study participants or how the instrument was described in published reports. Operational inconsistencies seemed to exist even within the most used standardized tool, the Menopause Rating Scale. The original article for the Menopause Rating Scale lists the item as “Heart discomfort (unusual awareness of heartbeat, heart skipping, heart racing, tightness)” [141, 142]. Most articles did not include all of the details in the parentheses in describing the item used for assessment and as noted above, did not report recall periods, and/or used varied response options. Conceptual and operational clarity cannot be reached until palpitations are well defined, and there is clarity surrounding which words should be used to describe the symptom.

Because symptoms are multidimensional [14], measures should assess multiple dimensions. Our finding that measures were mostly limited to assessing presence and severity is similar to findings from another review showing that cancer symptom measures are also limited to symptom presence and severity [143]. Multiple dimensions of palpitations have rarely been evaluated in the context of menopausal articles [12, 46, 82, 114, 124]. Multidimensional measures capture multiple characteristics and manifestations of symptoms compared to measures that focus on only one or two dimensions [144]. Assessing a greater number of symptom dimensions will provide a more detailed picture of the symptom profile and its complexity [145]. Measuring multiple palpitations dimensions can advance our understanding of the symptom and can help determine the efficacy and effectiveness of interventions [146].

Defining the concept of palpitations, including domain and subdomains, is a critical first step in measure development [147]. Because greater conceptual clarity can be reached by reviewing and synthesizing evidence from the literature and noting the field’s limitations [146], this review has helped to document where the field could move forward. In addition, talking with women about their palpitation symptom experience can help to generate items. Subsequently, receiving experts’ critique and review of such items will also strengthen measure development.

### Strengths and limitations of the review

Strengths of this review were the number of articles that we identified using multiple comprehensive databases which reduced the possibility of missing relevant articles. In addition, there were multiple authors involved at each stage of the review, which reduced the likelihood of subjectivity or inaccuracies.

The review also has some limitations. We only included English language articles, which may have led to the exclusion of some relevant articles. Choice of search terms is another limitation, which may have resulted in exclusion of some relevant articles. For example, the search picked up Obermeyer et al. [131], but not Obermeyer et al. [148]. However, the choice of search terms was difficult as there are no standard MeSH terms or other subject heading terms and no standardized measures for palpitations. We did not extract information on reliability and validity because these were not reported for the single item related to palpitations. We cannot derive a comprehensive definition of menopausal palpitations in this review since we aimed to explore how palpitations were measured. Nevertheless, we provided evidence of how the palpitations were measured and which symptom dimensions have and have not been measured.

## Conclusions

This review indicates that although palpitations have been assessed in menopausal women, nearly all assessments have been limited to single items. There was a lack of consistency on item wordings and response options, limited information on recall periods that were used, and a limited number and type of symptom dimensions that have been measured. The information in this review provides evidence for a need to create a multidimensional measure of menopausal palpitations. The review also serves as a cautionary tale for research on other menopausal symptoms because the measurement inconsistencies we identified are also problematic for all other menopausal symptoms assessed on these measures.

## Data Availability

Not applicable.
